# Pin vs plate fixation for metacarpal fractures: a meta-analysis

**DOI:** 10.1186/s13018-020-02057-y

**Published:** 2020-11-19

**Authors:** Xiangting Zhu, Hongwei Zhang, Jingying Wu, Shiwei Wang, Lin Miao

**Affiliations:** 1 Department of Orthopedics, Zaozhuang Hospital of Traditional Chinese Medicine, 2666 Taihangshan Road, Zaozhuang, 277000 Shandong People’s Republic of China; 2 Department of Emergency, Zaozhuang Hospital of Traditional Chinese Medicine, Zaozhuang, 277000 Shandong People’s Republic of China; 3 Department of Operation Room, Zaozhuang Hospital of Traditional Chinese Medicine, Zaozhuang, 277000 Shandong People’s Republic of China

**Keywords:** Metacarpal fractures, Percutaneous pin fixation, Open reduction with internal fixation, Meta-analysis

## Abstract

**Background:**

The differences in the clinical and functional outcomes of closed reduction and percutaneous pin fixation and open reduction with internal fixation (ORIF) using plate and screws have been systematically synthesized by one meta-analysis. With newer studies being published, an effort to update the earlier meta-analysis is necessary.

**Methods:**

Comprehensive searches were done systematically through PubMed, Scopus, CENTRAL (Cochrane Central Register of Controlled Trials), and Google scholar databases. Randomized controlled trials, quasi-experimental studies, prospective comparative non-randomized studies, and even studies reporting findings from retrospective chart review were eligible to be included. Statistical analysis was done using STATA version 13.0. GRADE assessment was done to assess the quality of pooled evidence.

**Results:**

A total of 9 studies were included. The pooled estimates did not suggest any significant differences in the disabilities of the arm, shoulder, and hand (DASH) score [WMD − 0.77; 95% CI, − 3.55, 2.00; *I*^2^ = 75.5%], range of movement (ROM) of the metacarpophalangeal joint (^o^) [WMD 4.44; 95% CI, − 4.19, 13.07; *I*^2^ = 86.0%], and grip strength [WMD − 4.63; 95% CI, − 14.52, 5.26; *I*^2^ = 86.9%] among the two intervention modalities. No difference was seen in the risk of complications between the two interventions (RR 0.93; 95% CI, 0.57, 1.53; *I*^2^ = 31.2%). For all the outcomes, the quality of pooled evidence was judged as low to very low.

**Conclusion:**

No significant long-term differences were noted in the functional outcomes suggesting that both these techniques are comparable. The choice of modality should be made based on the skills and preference of the surgeon and availability of resources.

**Supplementary Information:**

The online version contains supplementary material available at 10.1186/s13018-020-02057-y.

## Background

Emergency departments usually have a high inflow of patients with hand injuries, and metacarpal fractures represent around half (40%) of these hand injuries [1, 2]. Metacarpal fractures often comprise a large proportion of all hand fractures and fractures below the elbow, particularly in industrialized environments such as the USA [2–4]. Either accidental falls or direct impact trauma is responsible for most of these fractures. Clinical evidence shows that the neck of the metacarpal, the fifth metacarpal in particular, is the most affected [3]. The main goal of surgical management is to restore the bony shape, to enhance early mobilization, and to avoid functional impairment [3, 4].

Current metacarpal fracture management relies on data from individual studies that concentrate on a standalone modality. Given that there is a large difference in the fracture patterns and the underlying mechanism, it is difficult to perform controlled clinical trials [5, 6]. The two surgical modalities that have recently emerged for the management of metacarpal fractures that cannot be treated by casting alone are closed reduction and percutaneous pin fixation and open reduction with internal fixation (ORIF) using plate and screw [5, 6].

In terms of limited surgical exposure and feasibility of administration, pinning has an advantage [7, 8]. The use of plates and screws, on the other hand, offers direct fracture reduction and enables an early range of motion [7, 8]. Moreover, newer plates are smaller in size, allowing periosteal closure and potentially reducing adhesions compared to previously built plates [7, 8]. Due to variability in the sample population, surgical experience, and operational definitions of the outcomes considered, recent attempts to compare plate and pin fixation for metacarpal fractures have yielded mixed and equivocal results. Till date, the outcomes for these two treatment modalities have been systematically synthesized by one meta-analysis [9]. Through inclusion of four comparative studies and one secondary data review providing a sample of 222 patients, this meta-analysis found higher motion scores in subjects undergoing pinning for metacarpal fractures compared to ORIF with plate and screws. However, no significant differences were observed for functional scores, grip strength, radiographic parameters, and time to union [9]. With newer studies being published, an effort to update the earlier meta-analysis could shed more light on the comparative clinical and functional efficacy of the two treatment modalities. The present meta-analysis was conducted with the primary goal to perform a systematic literature search and conduct an updated meta-analysis of studies comparing plate and pin fixation of metacarpal fractures. The key outcomes considered were mostly functional outcomes and included disabilities of the arm, shoulder, and hand score (DASH score); percentage range of motion attained; and attained grip strength.

## Methods

### Search strategy

A comprehensive search was done systematically through PubMed, Scopus, CENTRAL, and Google scholar databases for papers published up to 15 January 2020. Free-text words and medical subject heading (MeSH) terms were used. Details of the search strategy have been provided in the supplementary document (Supplementary Table 1). The key aim was to identify studies that evaluated the clinical and functional efficacy of closed reduction and percutaneous pin fixation, in comparison to open reduction with internal fixation (ORIF) using plate and screws. Studies that reported relevant outcome measures of interest to this meta-analysis were potentially considered for inclusion.

### Selection criteria and methods

Two authors reviewed citations and selected studies. After removing the duplicates, screening of titles and abstracts was performed as a first step. Thereafter, a review of the full text of potential studies was done. Any discrepancies related to the inclusion of studies were resolved through detailed discussion among the study authors. Only those studies were selected for the meta-analysis that adequately suited the inclusion criteria. The bibliographic list of the identified studies and relevant reviews on the subject were examined for additional possible studies.

#### Inclusion criteria

Studies were eligible to be included in this meta-analysis if they compared the two treatment modalities with respect to functional and clinical outcomes. We did not have restrictions in terms of age, sex, and race of the participants. Randomized controlled trials, quasi-experimental studies, prospective comparative non-randomized studies, and even studies reporting findings from retrospective chart review were eligible to be included. The specific reason for being flexible enough to include non-randomized controlled trials was that there is a substantial variation in the patterns of metacarpal fractures and its underlying mechanism, and given this complexity, conducting controlled clinical trials are sometimes difficult [5, 6].

#### Exclusion criteria

Case reports and review articles were excluded.

### Data extraction and quality assessment

Extraction of relevant data from included studies was done by two authors independently, using a data extraction sheet. The following data from eligible studies were extracted: surname of the first author, year in which the study was published, geographical location where the study was done, design of the study, characteristics of the study subjects, study groups, and key findings of the study. Newcastle-Ottawa Quality Assessment Scale adapted for observational studies was used for quality assessment of included studies. For RCT, the methodological assessment was done using the Cochrane risk of bias assessment items [10, 11].

### Statistical analysis

Statistical analysis was done using STATA version 13.0 through “*metan*” command. Effect sizes were reported as weighted mean differences (WMD) for continuous outcomes and risk ratios (RR) for categorical variables. All estimates were reported with 95% confidence intervals (CI). Heterogeneity of effects was assessed and quantified by the *I*^2^. *I*^2^ value > 50% was considered to represent substantial heterogeneity [12]. In cases with substantial heterogeneity, the random effects model was used [12]. A *P* value of < 0.05 was considered statistically significant. Sub-group analysis was done based on the mean age of the study participants and the duration of follow-up, post-operatively, for outcome assessments. These were considered for sub-group analysis as they are important parameters that could help the surgeon to make a choice of the treatment modality. Publication bias was assessed using Egger’s test and visually inspected using funnel plots. The quality of the evidence generated was assessed using GRADE criteria and categorized as “high,” “moderate,” “low,” or “very low” [13]. PRISMA checklist was used for reporting of relevant items for this meta-analysis [14].

## Results

### Selection of articles, study characteristics, and quality of included studies

**Fig. 1:**
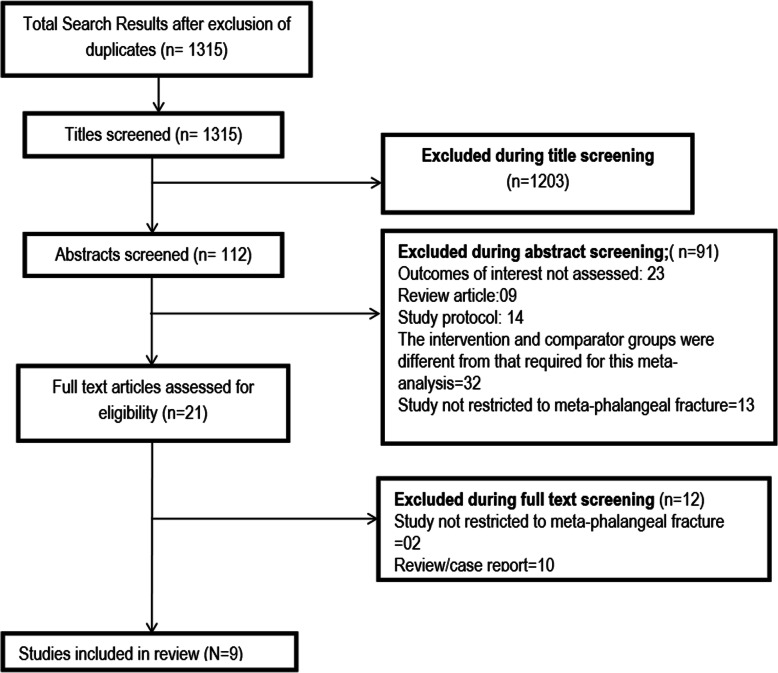
Selection process of the studies included in the review

A total of 1315 unique citations were obtained upon executing the search strategy in the PubMed, Scopus, CENTRAL (Cochrane Central Register of Controlled Trials), and Google scholar databases (Fig. 1). Out of these, 1203 were excluded based on title screening. Further, 91 citations were excluded after reading the abstract. Full text of the remaining 21 articles was reviewed. Out of these, 12 articles were excluded upon full-text review. The final number of included articles in this meta-analysis was 9 [15–23]. Table 1 presents the key characteristics of the included studies along with the key findings. All the included studies were non-randomized, except one by Pandey et al. [18] which was a randomized clinical trial. Two studies each were done in India, Japan, and the USA whereas one study each was done in France, Korea, and Israel. In all the included studies, majority of the study participants were males. The duration of follow-up for clinical and functional outcomes was 1 year or more in three studies whereas in the remaining 6 studies, outcomes were assessed within a year of surgery. The mean age of the study participants was > 30 years in 6 studies, and in the remaining three studies, it was ≤ 30 years. Supplementary Table 2 presents the findings of the quality assessment of included studies. All the included studies had low to moderate quality.

**Table 1:** Key details of the studies included in the meta-analysis

Author, year of publication	Country	Study design	Subjects	Intervention and control groups; point of assessment	Key outcome
Cha et al. (2019) [15]	Korea	Quasi-experimental	Patients with metacarpal fracture Mean (SD) age (years) Group 1, 37 (12) Group 2, 40 (11) Proportion of male subjects (56/69)—82%	Group 1 (mini-open antegrade intramedullary nailing) Group 2 (open reduction with internal fixation; ORIF) Point of assessment: clinical and functional outcomes were assessed at least 2 years after surgery	**Final shortening; mean (SD) in mm** Group 1 (*N* = 36), 0.3 (0.7) Group 2 (*N* = 33), 0.1 (0.5) **Final visual analogue score (VAS); mean (SD)** Group 1 (*N* = 36), 0.3 (0.6) Group 2 (*N* = 33), 0.3 (0.6) **Final DASH (disabilities of the arm, shoulder, and hand) score; mean (SD)** Group 1 (*N* = 36), 4 (3) Group 2 (*N* = 33), 6 (3) **Final range of movement (ROM) of the metacarpophalangeal joint (**^**o**^**); mean (SD)** Group 1 (*N* = 36), 84 (4) Group 2 (*N* = 33), 85 (3) **Final grip strength (% of the unaffected side); mean (SD)** Group 1 (*N* = 36), 94 (5) Group 2 (*N* = 33), 91 (5)
Dreyfuss et al. (2018) [16]	Israel	Non-randomized study	Adult patients operated for metacarpal shaft fractures Mean (range) age of participants (in years) Group 1, 27.5 (18–55) Group 2, 29.4 (18–57) All male subjects	Group 1 (pinning using Kirschner wire) Group 2 (open reduction with internal fixation with locking plates and screws) Point of assessment: clinical and functional outcomes were assessed at least 1 year after surgery	**Final shortening; mean (SD) in mm** Group 1 (*N* = 39), 1 (0.8) Group 2 (*N* = 29), 0 (0.0) **Final DASH score; mean (SD)** Group 1 (*N* = 39), 15.6 (8.8) Group 2 (*N* = 29), 10.5 (6.7) **Final range of movement (ROM) of the metacarpophalangeal joint (**^**o**^**); mean (SD)** Group 1 (*N* = 39), 71 (24.2) Group 2 (*N* = 29), 86 (12.5) **Final grip strength (% of the unaffected side); mean (SD)** Group 1 (*N* = 39), 83 (11.6) Group 2 (*N* = 29), 93 (13.8)
Vasilakis et al. (2019) [17]	USA	Retrospective chart review	Patients aged over 16 years with single digit, closed isolated extraarticular metacarpal fracture Mean (SD) age (years) Group 1, 37.9 (17.8) Group 2, 36.8 (16.1) Proportion of male subjects (49/70)—70%	Group 1 (closed reduction with percutaneous pinning) Group 2 (open reduction with internal fixation) Point of assessment: clinical and functional outcomes were assessed between 3 and 6 months post-operatively	**Final DASH score; mean (SD)** Group 1 (*N* = 44), 16.3 (7.1) Group 2 (*N* = 26), 18.7 (6.6) **Final range of movement (ROM) of the metacarpophalangeal joint (**^**o**^**); mean (SD)** Group 1 (*N* = 44), 90.8 (14.8) Group 2 (*N* = 26), 86.7 (20.6)
Pandey et al. (2018) [18]	India	RCT	Patients aged 16–60 years with closed shaft fracture of metacarpal Mean age (years) of the participants, 29.34 Proportion of male subjects (28/32)—87%	Group 1 (closed reduction with percutaneous pinning using Kirschner wire) Group 2 (open reduction with internal fixation) Point of assessment: clinical and functional outcomes were assessed at 2 years post-operatively	**Final DASH score; mean (SD)** Group 1 (*N* = 16), 32.98 (18.2) Group 2 (*N* = 16), 36.76 (16.6) **Final range of movement (ROM) of the metacarpophalangeal joint (**^**o**^**); mean (SD)** Group 1 (*N* = 16), 95.34 (24.9) Group 2 (*N* = 16), 95.82 (23.7)
Fujitani et al. (2012) [19]	Japan	Prospective quasi-randomized	Patients with displaced metacarpal neck fracture Mean (SD) age (years) of the participants, 31 (11) Group 1, 28 (13) Group 2, 33 (8) Proportion of male subjects (26/30)—87%	Group 1 (closed reduction with percutaneous pinning using Kirschner wire) Group 2 (open reduction with internal fixation) Point of assessment: clinical and functional outcomes were assessed within 1 year post-operatively	**Final shortening; mean (SD) in mm** Group 1 (*N* = 15), 1.5 (0.4) Group 2 (*N* = 15), 0.7 (0.5) **Final range of movement (ROM) of the metacarpophalangeal joint (**^**o**^**); mean (SD)** Group 1 (*N* = 15), 93 (23) Group 2 (*N* = 15), 78 (23) **Final grip strength (% of the unaffected side); mean (SD)** Group 1 (*N* = 15), 67 (18.3) Group 2 (*N* = 15), 86 (20.9)
Ozer et al. (2008) [20]	USA	Prospective quasi-randomized	Patients with closed, displaced extraarticular metacarpal fracture Mean age (range) (in years) of the participants Group 1, 25 (19–45) Group 2, 28 (19–47) Proportion of male subjects (35/52)- 67%	Group 1 (intramedullary nail fixation) Group 2 (plate screw fixation) Point of assessment: clinical and functional outcomes were assessed at 18–19 weeks (i.e., ~ 5 months) post-operatively	**Final shortening; mean (SD) in mm** Group 1 (*N* = 38), 3 (0.83) Group 2 (*N* = 14), 0 (0.0) **Final DASH score; mean (SD)** Group 1 (*N* = 38), 9.47 (4.2) Group 2 (*N* = 14), 8.07 (4.5) **Final range of movement (ROM) of the metacarpophalangeal joint (**^**o**^**); mean (SD)** Group 1 (*N* = 38), 91 (14) Group 2 (*N* = 14), 83 (23)
Facca et al. (2010) [21]	France	Prospective comparative non-randomized	Patients with closed, isolated, displaced 5th metacarpal neck fractures Mean age (in years) of the participants, 32.1 Proportion of male subjects (34/38)—90%	Group 1 (intramedullary K-wire fixation) Group 2 (locked plate screw fixation) Point of assessment: clinical and functional outcomes were assessed at a mean follow-up period of 3.3 months in group 1 and 4.8 months in group 2, post-operatively	**Final visual analogue score (VAS); mean (SD)** Group 1 (*N* = 20), 0.9 (1.02) Group 2 (*N* = 18), 0.94 (1.14) **Final DASH score; mean (SD)** Group 1 (*N* = 20), 9.8 (7.99) Group 2 (*N* = 18), 15.88 (7.47) **Final range of movement (ROM) of the metacarpophalangeal joint (**^**o**^**); mean (SD)** Group 1 (*N* = 20), 98 (4) Group 2 (*N* = 18), 74 (20) **Final grip strength (% of the unaffected side); mean (SD)** Group 1 (*N* = 20), 92.9 (20.6) Group 2 (*N* = 18), 88.4 (19.0)
Gupta et al. (2007) [22]	India	Prospective comparative non-randomized	Patients aged ≥ 14 years with closed, stable, extraarticular, non-avulsive metacarpal fracture Mean age (in years) of the participants, 35.6 The study was conducted among male subjects	Group 1 (reduction with percutaneous K-wire fixation) Group 2 (open/closed reduction with external fixation using locked plate/screw) Point of assessment: clinical and functional outcomes were assessed at 3 months, post-operatively	Total active range of motion was excellent in 42% (13/31) and good in 48.4% (15/31) of the patients in group 1. In group 2, in 42.8% patients, it was excellent and in 28.6% patients it was good. The observed differences were statistically non-significant. Total active range of motion was defined in terms of percent regained motion compared to the normal range of digital motion (i.e., 260°); excellent 85 to 100%; good 70–84%; fair 50–69%; and poor < 50%
Takigami et al. (2010) [23]	Japan	Retrospective	Patients operated for metacarpal fractures Mean (SD) age (in years) of the participants Group 1, 36 (21) Group 2, 45 (20) Proportion of male subjects (53/71)—75%	Group 1 (reduction with percutaneous K-wire fixation) Group 2 (reduction with low profile plate and screw) Point of assessment: clinical and functional outcomes were assessed at 6–13 months of being operated	Total active flexion (TAF) was 235° ± 38° in the low profile plate and screw group and 243° ± 22° in the K-wire group. This difference was not statistically significant. Total extension lag (TEL) was 12° ± 20° in the LPP group and 9° ± 12° in the K-wire group (not significant).

### Effect on DASH score

**Fig. 2:**
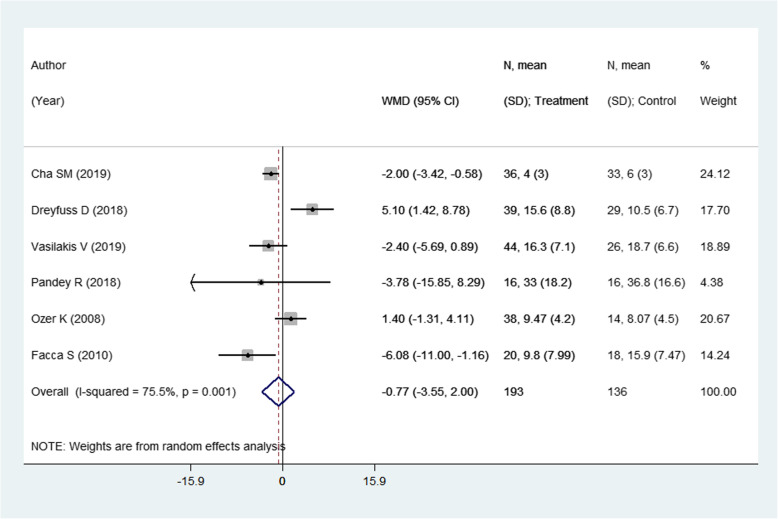
Comparison of pooled DASH scores among the two groups (i.e., pinning for metacarpal fractures compared to ORIF with plate and screws)

**Fig. 3:**
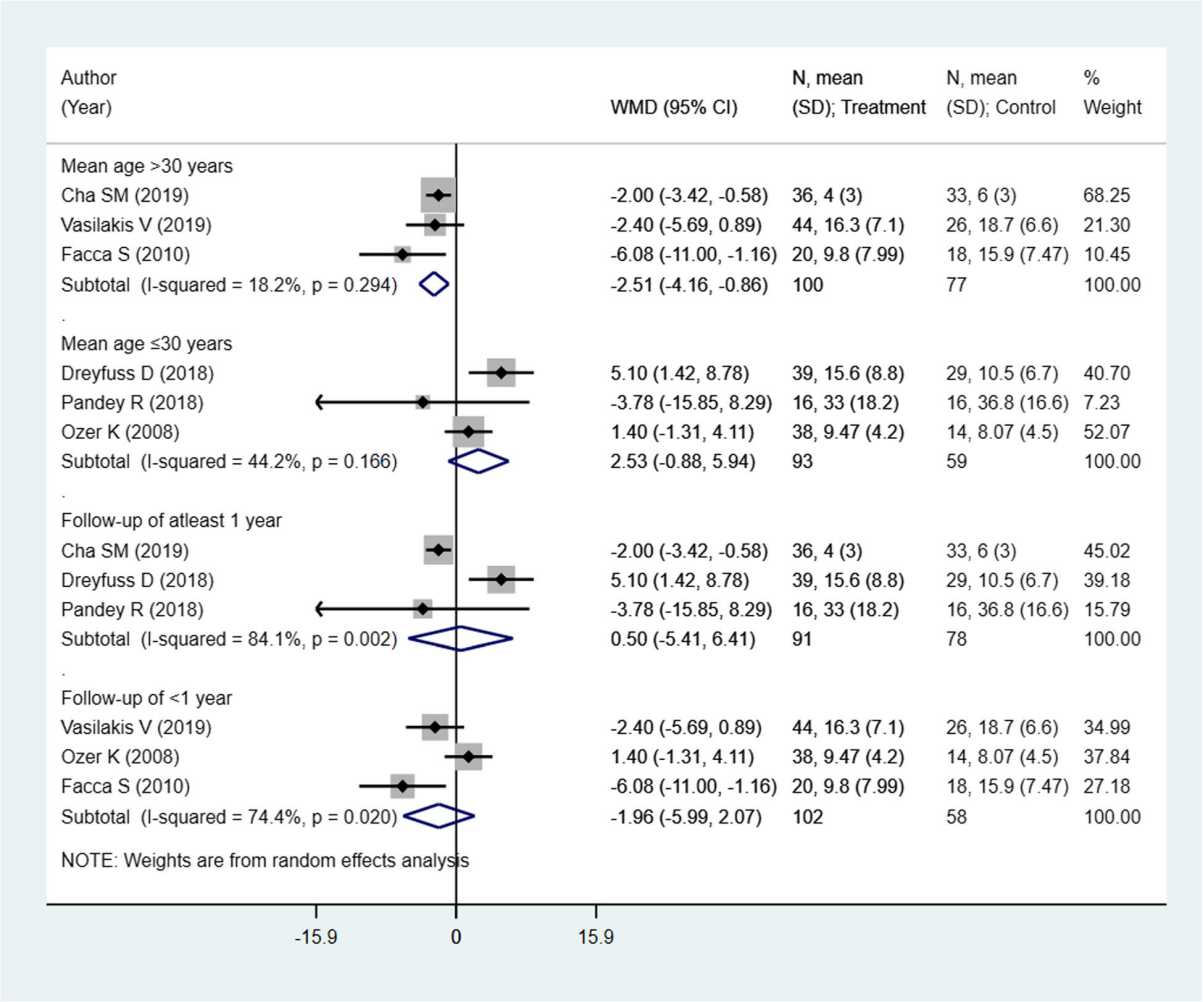
Comparison of pooled DASH scores among the two groups (i.e., pinning for metacarpal fractures compared to ORIF with plate and screws) by sub-groups based on the mean age of participants and duration of post-operative follow-up

There were 6 studies with 329 subjects reporting this outcome of interest. The pooled estimates did not suggest any significant differences in the DASH score among the two intervention modalities [weighted mean difference (WMD) − 0.77; 95% CI, − 3.55, 2.00; *I*^2^ = 75.5%] (Fig. 2). On sub-group analysis, pooling of studies with a mean age of the participants > 30 years showed lower DASH scores in those receiving pinning for metacarpal fractures compared to those undergoing ORIF with plate and screws [WMD − 2.51; 95% CI, − 4.16, − 0.86; *I*^2^ = 18.2%) (Fig. 3). The findings were not significant in any other sub-groups. There was no evidence of publication bias (*P* value = 0.83). The funnel plot is presented as Supplementary Figure 1. The overall quality of evidence was judged as “low” according to GRADE assessment (Table 2).

**Table 2:** Quality of evidence according to the GRADE criteria

Outcomes	Number of studies (design); no. of participants	Effect size (95% CI)	Characteristics of the included studies					
			Risk of bias^a^	Inconsistency	Indirectness^b^	Imprecision^c^	Publication bias	Overall GRADE quality score
Disabilities of the arm, shoulder, and hand score (DASH)	6 (5 observational; 1 RCT); *n* = 329	WMD − 0.77 (− 3.55, 2.00)	Serious	Not serious	Serious	Not serious	Undetected	⨁⨁◯◯ Low
Range of movement (ROM) at the meta-carpo-phalangeal joint	7 (6 observational; 1 RCT); *n* = 359	WMD 4.44 (− 4.19, 13.07)	Serious	Not serious	Serious	Not serious	Undetected	⨁⨁◯◯ Low
Grip strength	4 (4 observational); *n* = 205	WMD − 4.63 (− 14.52, 5.26)	Serious	Not serious	Serious	Serious^c^	Undetected	⨁◯◯◯ Very low
Limb shortening (in mm) on radiography	4 (4 observational); *n* = 219	WMD 1.25 (0.03, 2.47)	Serious	Not serious	Serious	Serious ^c^	Undetected	⨁◯◯◯ Very low
Visual analogue score (VAS)	2 (2 observational); *n* = 107	WMD − 0.01 (− 0.27, 0.26)	Serious	Not serious	Serious	Serious^c^	Undetected	⨁◯◯◯ Very low
Complication rates	8 (7 observational; 1 RCT); *n* = 439	RR 0.93 (0.57, 1.53)	Serious	Not serious	Serious	Not serious	Undetected	⨁⨁◯◯ Low

^a^Majority of the studies included were observational in design

^b^Studies were done in different geographic settings. Further, studies differed in the age of the participants and the duration of follow-up post-operatively

^c^Criteria for optimal information size (OIS) not met and the 95% CI overlap no effect and includes important benefit and harm

### Effect on the Range of Movement (ROM) of the metacarpophalangeal joint (°)

**Fig. 4:**
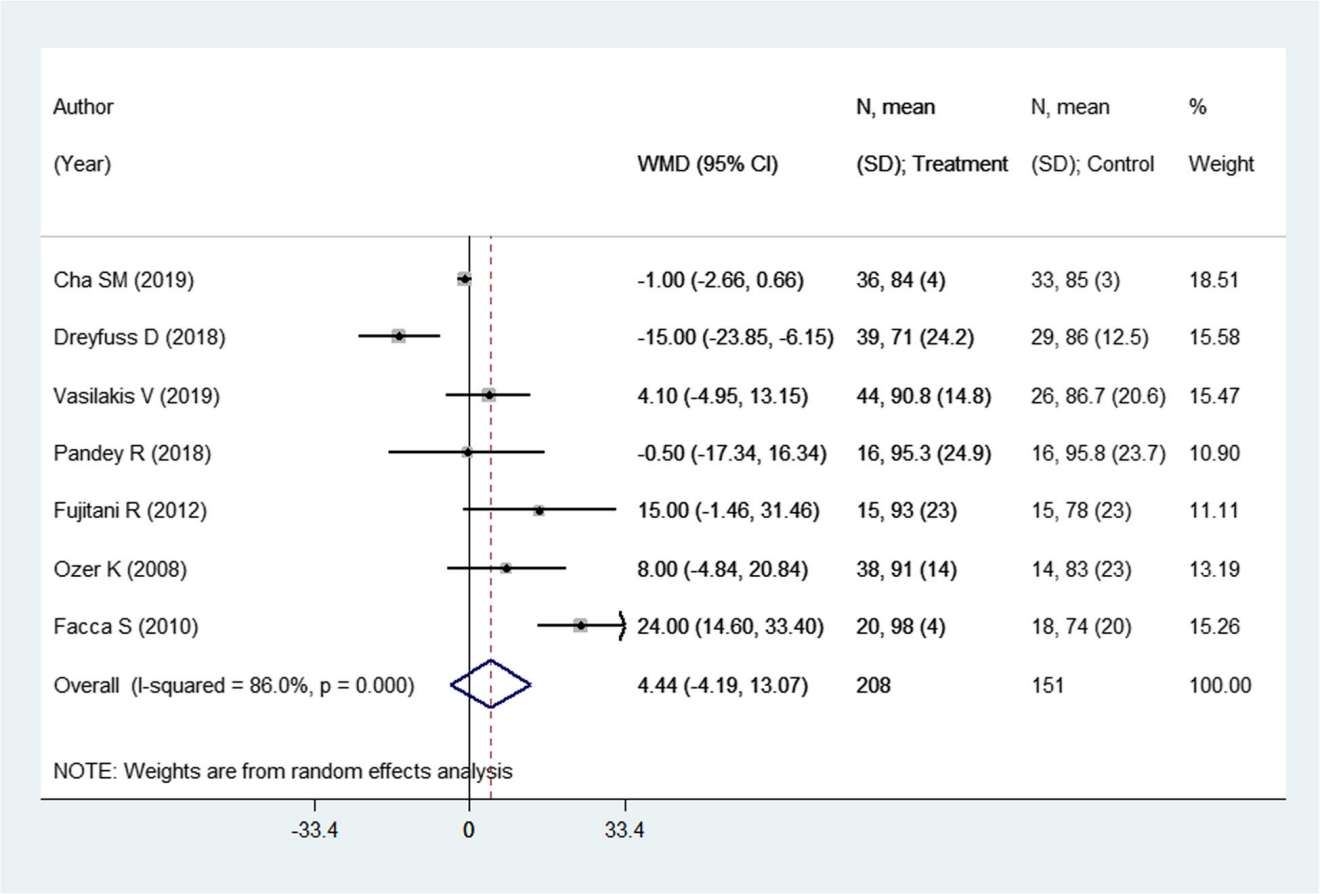
Comparison of pooled range of movement (ROM) of the metacarpophalangeal joint (°) among the two groups (i.e., pinning for metacarpal fractures compared to ORIF with plate and screws)

**Fig. 5:**
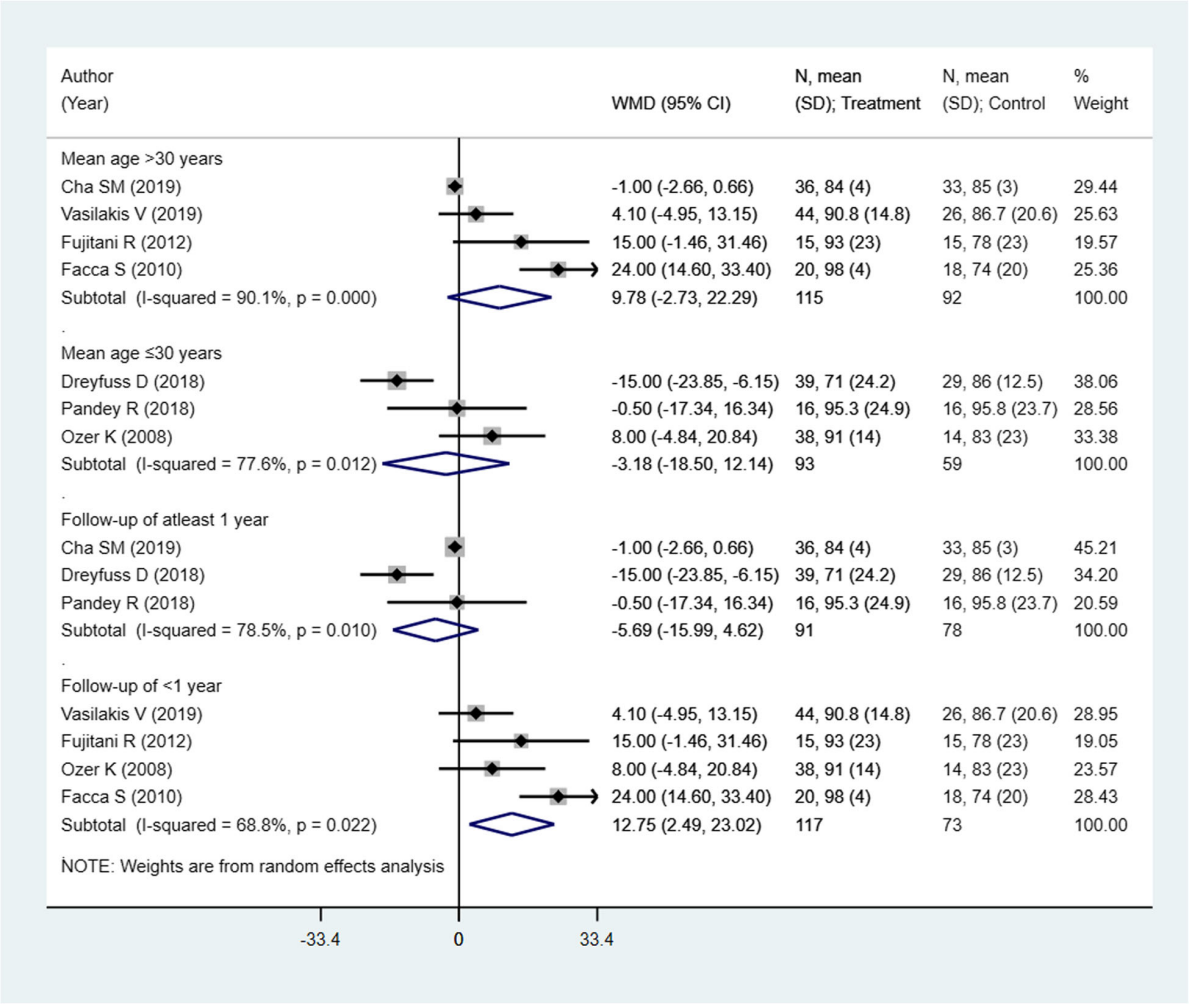
Comparison of pooled range of movement (ROM) of the metacarpophalangeal joint (°) among the two groups (i.e., pinning for metacarpal fractures compared to ORIF with plate and screws) by sub-groups based on the mean age of participants and duration of post-operative follow-up

This outcome was reported in 7 studies with an overall sample size of 359. The pooled estimates suggest a similar degree of attained range of movement of the metacarpophalangeal joint in the two modalities (WMD 4.44; 95% CI, − 4.19, 13.07; *I*^2^ = 86.0%) (Fig. 4). On sub-group analysis, pooling of studies with shorter follow-up period, i.e., of less than 1 year, showed a better range of movement (ROM) in those receiving pinning for metacarpal fractures compared to those undergoing ORIF with plate and screws [WMD 12.75; 95% CI, 2.49, 23.02; *I*^2^ = 68.8%) (Fig. 5). The findings were not significant in any other sub-groups. There was no evidence of publication bias (*P* value = 0.38). The funnel plot is presented as Supplementary Figure 2. The overall quality of evidence was judged as “low” according to GRADE assessment (Table 2).

### Effect on grip strength (as percentage of the unaffected side)

**Fig. 6:**
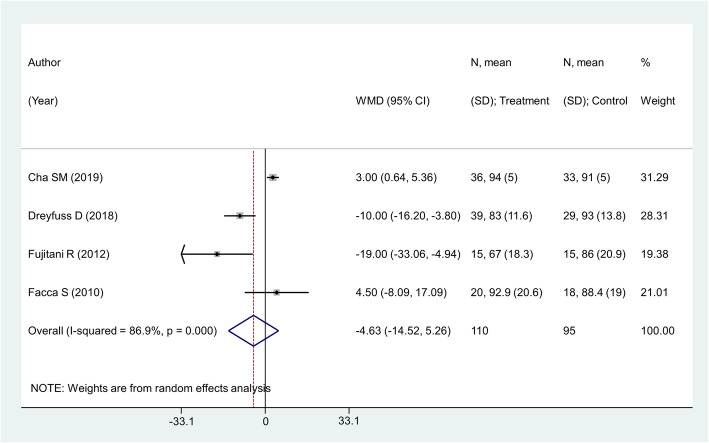
Comparison of pooled grip strength (as percentage of the unaffected side) among the two groups (i.e., pinning for metacarpal fractures compared to ORIF with plate and screws)

**Fig. 7:**
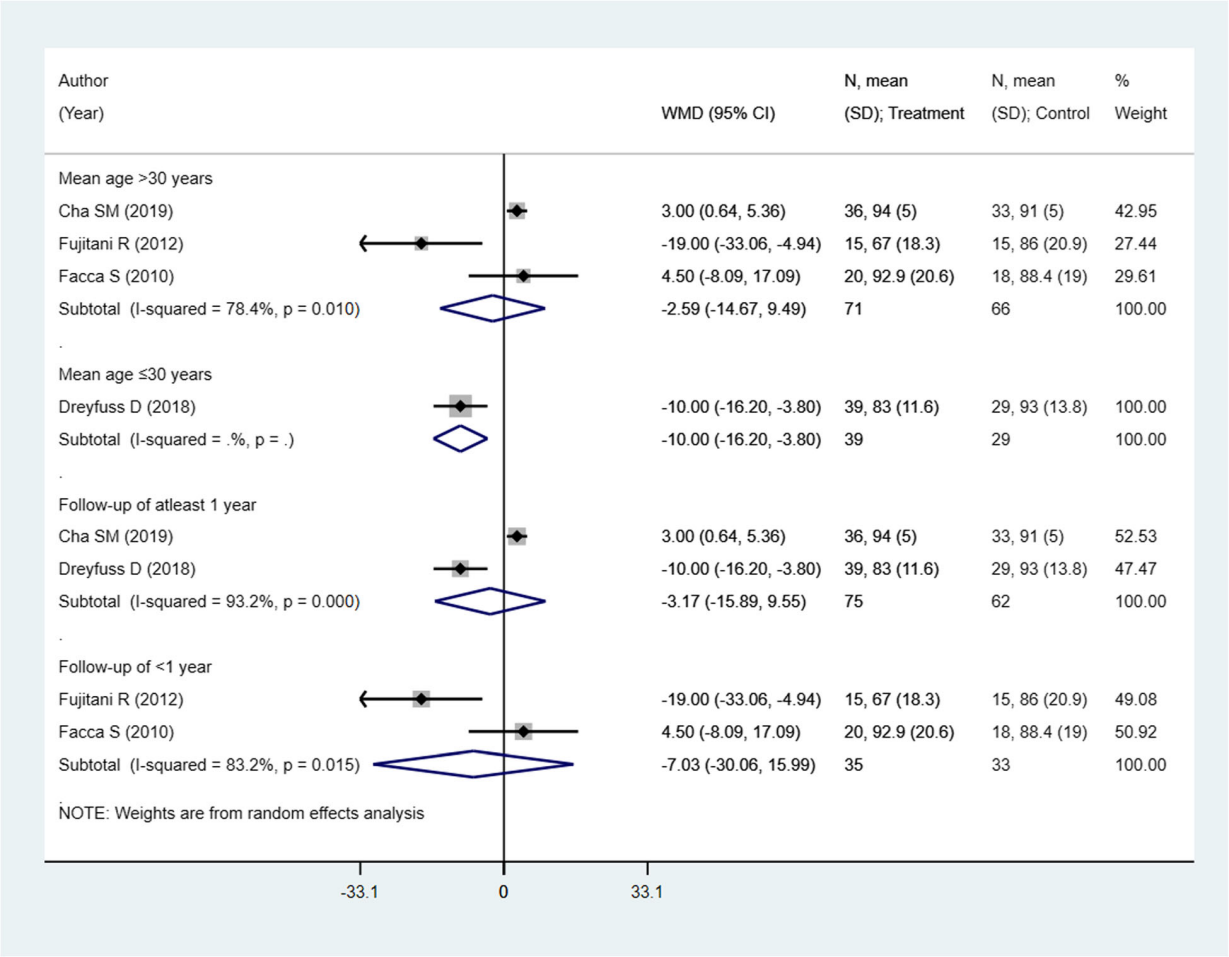
Comparison of pooled grip strength (as percentage of the unaffected side) among the two groups (i.e., pinning for metacarpal fractures compared to ORIF with plate and screws) by sub-groups based on the mean age of participants and duration of post-operative follow-up

There were 4 studies with 205 subjects reporting this outcome of interest. The pooled estimates did not suggest any significant differences in the grip strength among the two intervention modalities [weighted mean difference (WMD) − 4.63; 95% CI, − 14.52, 5.26; *I*^2^ = 86.9%) (Fig. 6). No significant differences between the two comparison groups in any of the sub-groups were noted, except for the sub-group with the mean age of participants ≤ 30 years. However, there was only one study in this sub-group with a small sample size of 68 (Fig. 7). There was no evidence of publication bias (*P* value = 0.31). The funnel plot is presented as Supplementary Figure 3. The overall quality of evidence was judged as “very low” according to GRADE assessment (Table 2).

### Effect on limb shortening (in mm) assessed by radiography

**Fig. 8:**
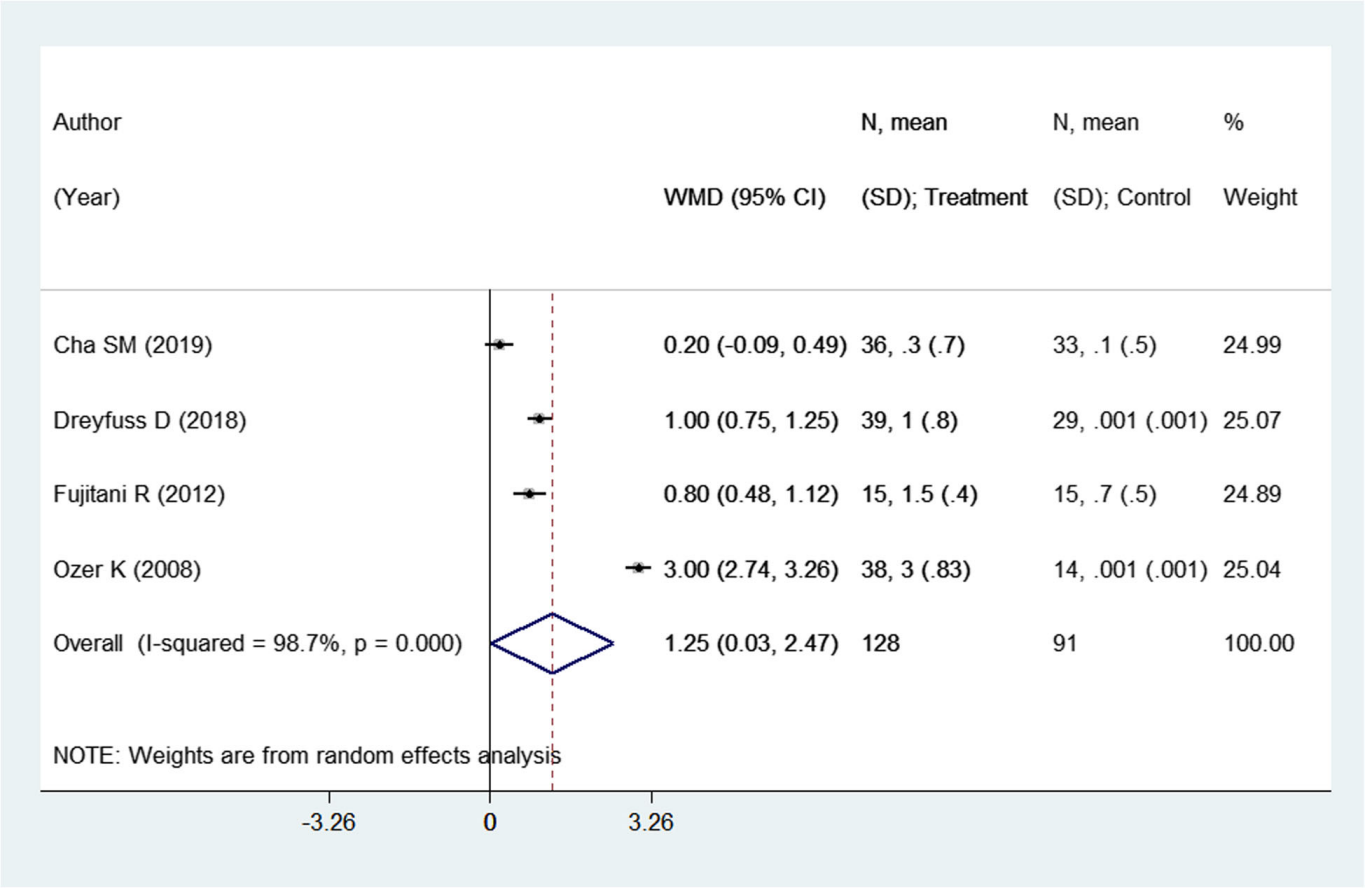
Comparison of limb shortening (in mm) assessed by radiography among the two groups (i.e., pinning for metacarpal fractures compared to ORIF with plate and screws)

**Fig. 9:**
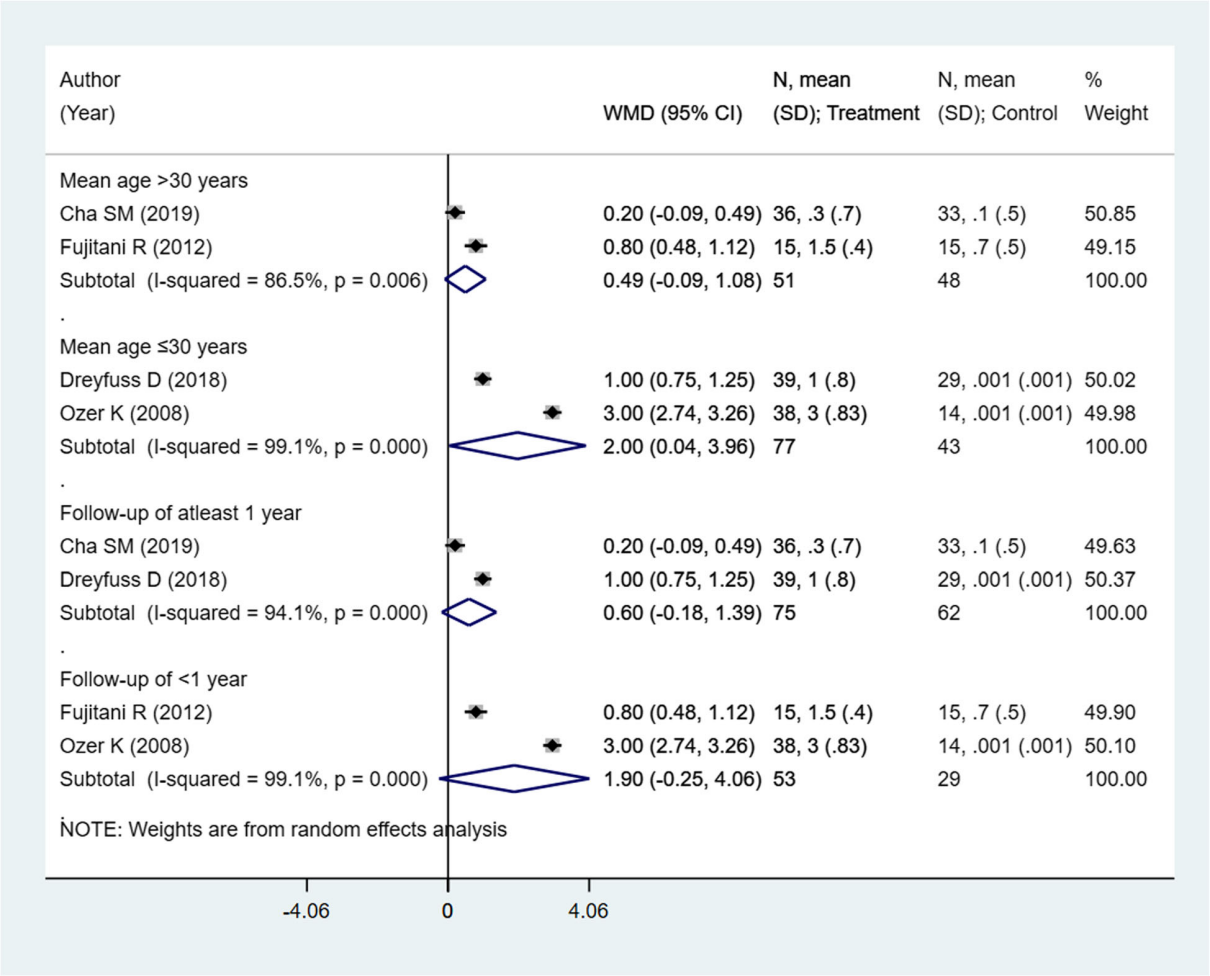
Comparison of limb shortening (in mm) assessed by radiography among the two groups (i.e., pinning for metacarpal fractures compared to ORIF with plate and screws) by sub-groups based on the mean age of participants and duration of post-operative follow-up

There were 4 studies with 219 subjects reporting this outcome of interest. The pooled estimates indicate significant differences in the limb shortening, assessed radiologically, among the two intervention modalities. Patients undergoing closed reduction and percutaneous pin fixation had comparatively higher shortening compared to those receiving open reduction with internal fixation (ORIF) using plate and screws [weighted mean difference (WMD) 1.25; 95% CI, 0.03, 2.47; *I*^2^ = 98.7%) (Fig. 8). On sub-group analysis, pooling of studies with a mean age of the participants ≤ 30 years showed comparatively higher shortening in those receiving pinning for metacarpal fractures compared to those undergoing ORIF with plate and screws [WMD 2.00; 95% CI, 0.04, 3.96; *I*^2^ = 99.1%) (Fig. 9). The findings were not significant in any other sub-groups. There was no evidence of publication bias (*P* value = 0.91). The funnel plot is presented as Supplementary Figure 4. The overall quality of evidence was judged as “very low” according to GRADE assessment (Table 2).

### Effect on Visual Analogue Scale (VAS) pain score

**Fig. 10:**
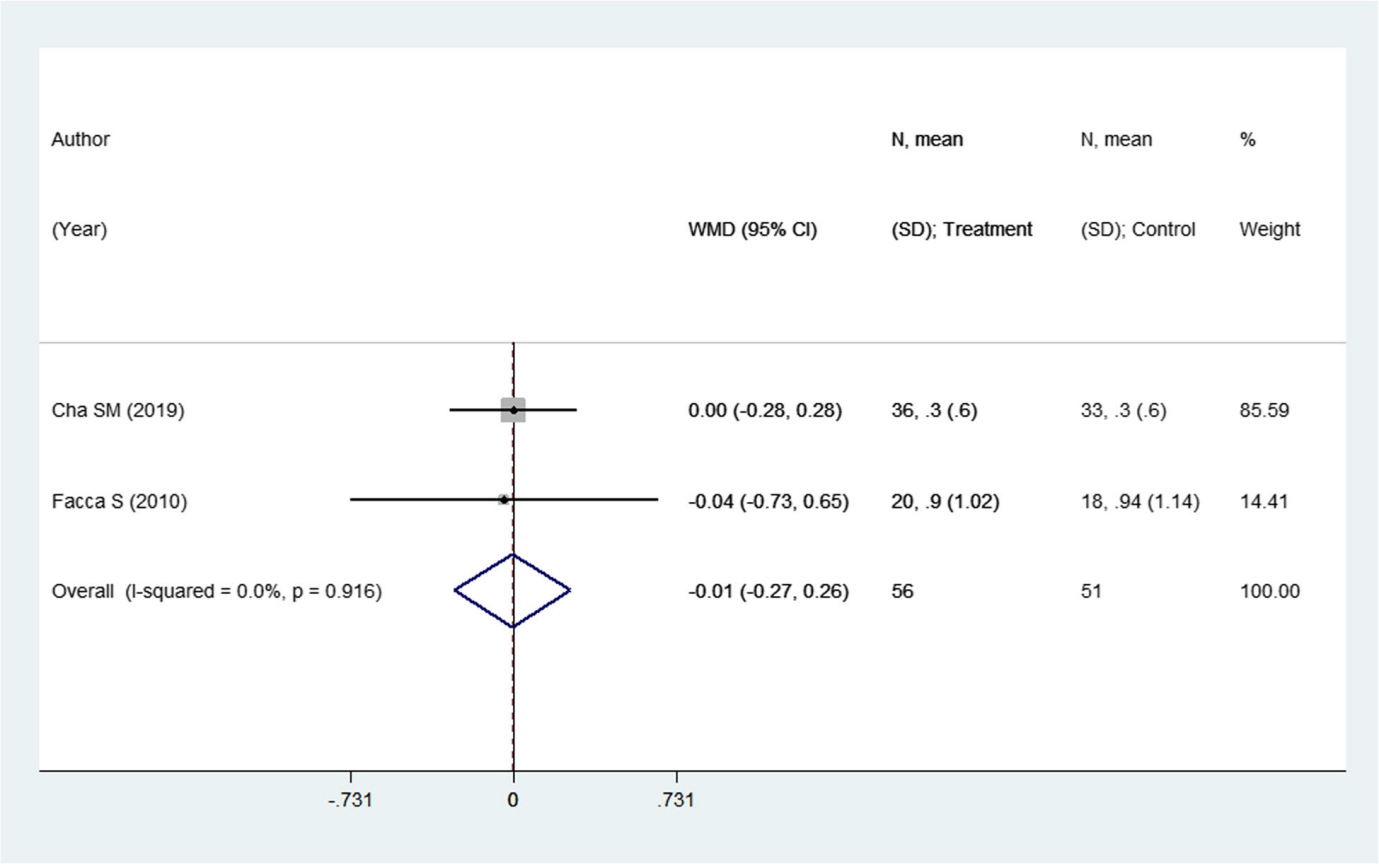
Comparison of visual analogue scale (VAS) pain score among the two groups (i.e., pinning for metacarpal fractures compared to ORIF with plate and screws)

This outcome was reported in 2 studies with an overall sample size of 107. The pooled estimates suggest no significant differences in the pain score in the two modalities (WMD − 0.01; 95% CI, − 0.27, 0.26; *I*^2^ = 0.0%) (Fig. 10). As there were only two studies reporting this outcome, publication bias and funnel plot could not be generated.

### Complications

**Fig. 11:**
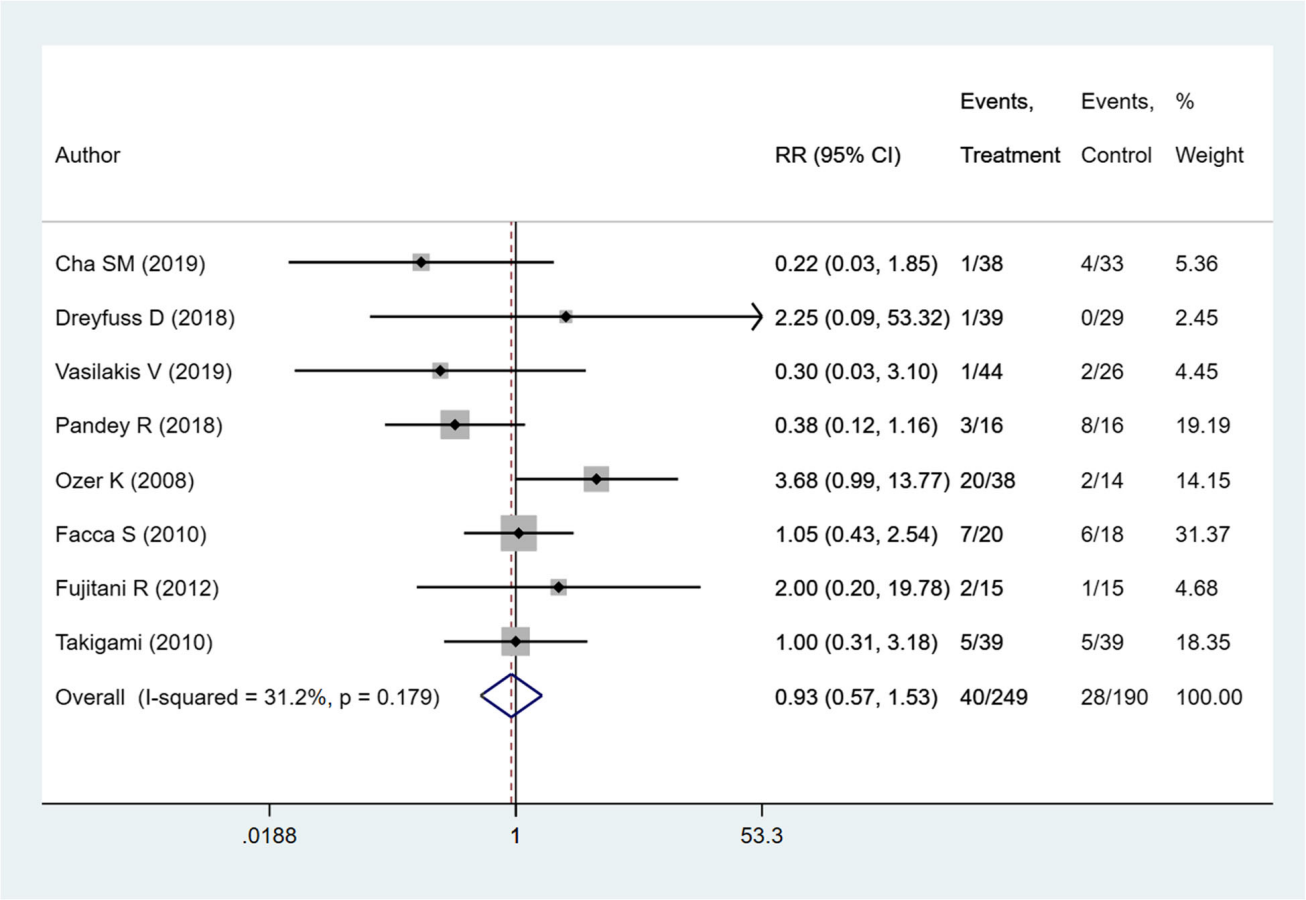
Comparison of complication rates among the two groups (i.e., pinning for metacarpal fractures compared to ORIF with plate and screws)

Details of complications reported by included studies are presented in Table 3. Meta-analysis indicated no significant difference in the risk of complications between the two intervention modalities (RR 0.93; 95% CI, 0.57, 1.53; *I*^2^ = 31.2%, *P* = 0.179) (Fig. 11). The overall quality of evidence was judged as “low” according to GRADE assessment (Table 2).

**Table 3:** Data on time to union, complications, and residual angulation at the fracture site from the included studies

Studies	Time to union		Complications with the number of patients		Residual angulation at the fracture site (degrees)	
	Pin group	Plate group	Pin group	Plate group	Pin group	Plate group
Cha et al. [15]	NR	NR	Superficial infection, 1	Extensor lag, 4	0.8 ± 1	0.6 ± 0.1
Dreyfuss et al. [16]	50 (28–286)^a^ days	59 (37–105)^a^ days	Work-related fracture, 1	Nil	AP, 1 (0–11) Lateral, 1 (0–9)	AP, 0 Lateral, 0
Vasilakis et al. [17]	NR	NR	Stiffness requiring extensor tenolysis, 1	Non-union, 1 Hardware removal, extensor tenolysis, 1	NR	NR
Pandey et al. [18]	3 months	3 months	Infection. 1 Malunion, 1 Transient numbness, 1	Transient numbness, 4 Prominent implant and impingement at terminal motion, 4	NR	NR
Fujitani et al. [19]	NR	NR	Transient neuritis of the dorsal ulnar nerve, 1 Extensor tendon rupture, 1	Transient neuritis of dorsal ulnar nerve, 1	Palmar tilt, 16 (NR) Lateral tilt, 16 (NR)	Palmar tilt, 10 (NR) Lateral tilt, 10 (NR)
Ozer et al. [20]	5.4 (4–8) weeks	5.2 (4–7) weeks	Loss of reduction, 5 Hardware removal, 15	Hardware removal, 2	AP, 2(0–10) Lateral, 8 (0–25)	AP, 0 Lateral, 0
Facca et al. [21]	NR	NR	Wire migration. 3 Neurological lesions. 3 Aesthetic blemish due to callus. 1	Hardware removal. 3 Delayed consolidation. 2 Instability of site. 1	NR	NR
Gupta et al. [22]	NR	NR	NR	NR	NR	NR
Takigami et al. [23]	1.6 ± 0.6^a^ months	2.6 ± 1.6^a^ months	Superficial infection, 1 Soreness of pin site, 4	Screw breakage, 3 Screw loosening, 2	NR	NR

Data on time to union and residual angulation at fracture site presented as mean (range) or mean ± standard deviation

*AP* anteroposterior, *NR* not reported

^s^Statistical significant difference reported between the two groups

### Time to union and residual angulation

Due to the limited availability of data, only a descriptive analysis was carried out for these variables (Table 3). A total of four studies reported time to radiographic union with two studies reporting no statistically significant difference between the two fixation techniques while the remaining two reported significantly earlier healing in the pin fixation group. The four studies reporting radiographic residual angulation of the fracture site did not report any statistically significant difference between the two groups.

## Discussion

The present study was conducted with the intention to perform a systematic literature search and conduct a meta-analysis of studies comparing plate and pin fixation of metacarpal fractures with respect to functional outcomes such as DASH score, percentage range of motion attained, and attained grip strength. We did not find any significant differences in these primary outcomes among the two treatment modalities. No differences were found in the pain scores as well. However, we did find that patients undergoing closed reduction and percutaneous pin fixation had comparatively higher shortening on radiological assessment compared to those receiving open reduction with internal fixation (ORIF) using plate and screws, and this was most prominent in study subjects younger than 30 years.

Our findings are similar to the previous meta-analysis by Melamed et al. [9]. The earlier meta-analysis included 5 studies, and our review included 4 additional studies. In the previous meta-analysis, the included studies assessed outcomes within a year of surgery whereas in the current meta-analysis, 3 out of 4 additional studies assessed outcomes at > 1 year post-operatively. This is the advantage of our meta-analysis over the previous one. Our study presents pooled evidence on long-term effects of the two treatment modalities on clinical and functional outcomes. Similar to the previous meta-analysis, we have shown a better range of movement (ROM) within 1 year of post-operative period in those receiving pinning for metacarpal fractures compared to those undergoing ORIF with plate and screws. However, we have additionally shown that in the longer course (i.e., at > 1 year post-operatively), there are no statistically significant differences in the ROM between the two treatment modalities. The findings that functional scores and grip strength were not significantly different between the two groups corroborated with the findings of the earlier meta-analysis [9].

The decision to opt for either closed reduction and percutaneous pin fixation or open reduction with internal fixation (ORIF) using plate and screws should largely depend on the characteristics of the fracture, associated additional injuries, surgical skills of the treating doctor, and resources available [6, 8, 24]. With the continuous evolvement and improvement in the design of the implants for ORIF, the indications for its usage in different types of metacarpal fractures have widened and the success rates have also improved [8, 24]. Specially with the introduction of locking screws, the stability of the plates has improved tremendously. One of the important factors influencing the decision of pin vs plate fixation of metacarpal fractures is the accuracy of reduction required based on the fracture pattern. Spiral or oblique shaft fractures have a higher incidence of rotational deformity as compared to other fracture types. Malrotation which such fracture patterns can be better reduced with plate fixation [16]. While this outcome was not assessed in a meta-analysis in our review, on descriptive analysis, two of the included studies reported a complete absence of any residual angulation after plate fixation which was not the case with pin fixation [16, 20]. Given that plate fixation is performed under direct vision, it is plausible that more accurate reduction can be achieved with this technique. According to Vasilakis et al. [17], plate fixation is a viable mode of management in a variety of fracture patterns like transverse shaft fractures, spiral/oblique fractures, and fractures with delayed presentation owing to this advantage. However, the large exposure and striping of the periosteum required for plating may also affect radiographic healing time. Two of the included studies reported a longer duration of union in the plating group. Nevertheless, in clinical practice, plate fixation allows for an early return to function and better patient satisfaction and may be preferred when early mobilization is needed by the patient [16]. Having said this, the rates of complications were not found to be different between the two techniques. Our meta-analysis failed to demonstrate any increased risk of complications with ORIF considering the invasive nature of the procedure. However, it should be noted that there was heterogeneity in the types of complications pooled for the analysis and meta-analysis per complication could not be carried out due to the limited data.

For most of the outcomes in this meta-analysis, we used the random effects model as the heterogeneity was high. Eight of out nine studies included in this meta-analysis were observational. Further, studies had a limited sample size. Studies with small sample size are often met with the limitation of lack of generalizability of the findings. These could be reasons contributing to the low and very low quality of evidence observed in this meta-analysis. Consequently, there is a need for large studies, preferably randomized controlled trials, to conclusively establish the comparative efficacy of the two treatment modalities in the management of metacarpal fractures. An important point to note is that all the studies did not use identical implants in the study groups, and therefore, mere categorization into pinning and plating group is nothing but overt oversimplification. It should also be noted that the studies had different follow-up periods after which the clinical and functional outcomes were assessed. While we did a sub-group analysis based on the duration of follow-up, it would have been better if studies were somewhat homogenous in their follow-up periods.

## Conclusion

The meta-analysis provides updated pooled evidence on the comparative effectiveness of closed reduction and percutaneous pin fixation and open reduction with internal fixation (ORIF) using plate and screws. The lack of significant long-term differences in the functional outcomes suggests that both these techniques are comparable. Furthermore, the risk of complications was not significantly different with the two interventions. Consequently, the choice of treatment modality should be governed by the skills and preference of the surgeon and availability of resources. In order to conclusively tease out the differences in clinical and functional outcomes, if any, between these treatment modalities, future studies (preferably randomized controlled trials) should be done with a larger sample size.

## Supplementary Information

**Additional file 1:** **Supplemental Table 1.** Search strategy for identification of studies to be included in the review. **Supplementary Table 2 A.** Author’s judgements about study quality using the adapted Ottawa-Newcastle Risk of Bias Assessment for Observational Studies. **Supplementary Table 2 B.** Author’s judgements about risk of bias for the randomized controlled trial study based on Cochrane risk of bias assessment items. **Supplementary Figure 1.** Funnel plot for publication bias with respect to comparison of pooled DASH scores among the two groups (i.e. pinning for metacarpal fractures compared to ORIF with plate and screws). **Supplementary Figure 2.** Funnel plot for publication bias with respect to comparison of pooled range of movement (ROM) of the metacarpophalangeal joint (^o^) among the two groups (i.e. pinning for metacarpal fractures compared to ORIF with plate and screws). **Supplementary Figure 3.** Funnel plot for publication bias with respect to comparison of pooled grip strength (as % of the unaffected side) among the two groups (i.e. pinning for metacarpal fractures compared to ORIF with plate and screws). **Supplementary Figure 4.** Funnel plot for publication bias with respect to comparison of limb shortening (in mm) assessed by radiography among the two groups (i.e. pinning for metacarpal fractures compared to ORIF with plate and screws).

## Data Availability

The datasets used and/or analyzed during the current study are available from the corresponding author on reasonable request.

## Abbreviations

ORIF: Open reduction with internal fixation
DASH: Disabilities of the arm, shoulder, and hand
ROM: Range of movement
WMD: Weighted mean differences
CI: Confidence intervals
