# Supplementary material for: Pin vs plate fixation for metacarpal fractures: a meta-analysis
Source: J Orthop Surg Res. 2020 Nov 19;15:542. doi: 10.1186/s13018-020-02057-y (PMC7678208; doi:10.1186/s13018-020-02057-y)
Supplement: Supplementary file 1 — Additional file 1: Supplemental Table 1. Search strategy for identification of studies to be included in the review. Supplementary Table 2 A. Author’s judgements about study quality using the adapted Ottawa-Newcastle Risk of Bias Assessment for Observational Studies. Supplementary Table 2 B. Author’s judgements about risk of bias for the randomized controlled trial study based on Cochrane risk of bias assessment items. Supplementary Figure 1. Funnel plot for publication bias with respect to comparison of pooled DASH scores among the two groups (i.e. pinning for metacarpal fractures compared to ORIF with plate and screws). Supplementary Figure 2. Funnel plot for publication bias with respect to comparison of pooled range of movement (ROM) of the metacarpophalangeal joint (o) among the two groups (i.e. pinning for metacarpal fractures compared to ORIF with plate and screws). Supplementary Figure 3. Funnel plot for publication bias with respect to comparison of pooled grip strength (as % of the unaffected side) among the two groups (i.e. pinning for metacarpal fractures compared to ORIF with plate and screws). Supplementary Figure 4. Funnel plot for publication bias with respect to comparison of limb shortening (in mm) assessed by radiography among the two groups (i.e. pinning for metacarpal fractures compared to ORIF with plate and screws). [file 13018_2020_2057_MOESM1_ESM.doc]

**SUPPLEMENTARY TABLES AND FIGURES**

**Supplemental table 1. Search strategy for identification of studies to be included in the review**

| **Search strategy**  #1 (intramedullary wire OR open reduction with internal fixation OR closed reduction with pin fixation OR K-wire OR locking plates)  #2 (metacarpal fracture OR hand fracture OR metacarpophalangeal fracture)  #3 (Randomized controlled trial OR Controlled clinical trial OR prospective OR follow up OR retrospective OR observational OR quasi-experimental OR quasi-randomized)  #4 (#1 AND #2 AND #3)  #5 (Addresses[ptyp] OR Autobiography[ptyp] OR Bibliography[ptyp] OR Biography[ptyp] OR pubmed books[filter] OR Case Reports[ptyp] OR Congresses[ptyp] OR Consensus Development Conference[ptyp] OR Directory[ptyp] OR Duplicate Publication[ptyp] OR Editorial[ptyp] OR Systematic reviews OR Meta analysis OR Festschrift[ptyp] OR Guideline[ptyp] OR In Vitro[ptyp] OR Interview[ptyp] OR Lectures [ptyp] OR Legal Cases[ptyp] OR News[ptyp] OR Newspaper Article[ptyp] OR Personal Narratives [ptyp] OR Portraits[ptyp] OR Retracted Publication[ ptyp] OR Twin Study[ptyp] OR Video-Audio Media[ptyp])  #6 (#4 NOT #5) |
| --- |

**Supplementary table 2 A. Author’s judgements about study quality using the adapted Ottawa-Newcastle Risk of Bias Assessment for Observational Studies**

|  | Cha SM et al | Dreyfuss D et al | Vasilakis V et al | Fujitani R et al | Ozer K et al | Facca S et al | Gupta R et al | Takigami H et al |
| --- | --- | --- | --- | --- | --- | --- | --- | --- |
| Representativeness/appropriateness of participant selection  Random or consecutive recruitment=Y  Convenience sample=N  Not reported or unclear | Y | Y | Y | Y | Y | Y | Y | Y |
| Control for baseline differences in cohorts  Similarity of groups at baseline or adjustment in analyses=Y  No attempt to control or adjust=N  Not reported=NR | Y | Y | Y | Y | Y | Y | Y | Y |
| Loss to follow-up  Explanation provided for loss of participants and/or intention to treat=Y  No explanation =N | N | N | N | Y | N | N | Y | N |
| Masking of exposure to outcomes assessor  Description of masking=Y  No masking or no description =N | N | N | N | N | N | N | N | N |
| Ascertainment of condition  Description of ascertainment/diagnostic criteria=Y  No description or patient self-report=N | Y | Y | Y | Y | Y | Y | Y | N |
| Documentation of other treatment modalities  Documentation=Y  No documentation=N | Y | Y | Y | Y | Y | Y | Y | Y |
| Extent to which valid outcomes are described  Adequate description of outcome=Y  Insufficient detail regarding outcome or follow-up time=N | Y | Y | Y | Y | Y | Y | Y | Y |
| Prespecification of harms, mode of harms collection  Description of a list of harms assessed or monitoring=Y  No such description or passive harms collection=N  No adverse events reported=NA | Y | N | N | Y | N | Y | Y | N |
| Financial Conflict of interest (COI)  Funding source reported=Y  Funding source not reported=N | Y | Y | Y | Y | Y | Y | Y | Y |

**Supplementary table 2 B. Author’s judgements about risk of bias for the randomized controlled trial study based on Cochrane risk of bias assessment items**

| **Included studies** | **Random sequence generation** | **Allocation concealment** | **Blinding of participants** | **Blinding of personnel** | **Blinding of outcome assessment team** | **Attrition bias** | **Other bias** |
| --- | --- | --- | --- | --- | --- | --- | --- |
| Pandey R et al | + | ? | + | + | + | ? | None identified |

+ denotes presence; ? denotes unclear

**Supplementary Figure 1.** Funnel plot for publication bias with respect to comparison of pooled DASH scores among the two groups (i.e. pinning for metacarpal fractures compared to ORIF with plate and screws)

**Supplementary Figure 2.** Funnel plot for publication bias with respect to comparison of pooled range of movement (ROM) of the metacarpophalangeal joint (^o^) among the two groups (i.e. pinning for metacarpal fractures compared to ORIF with plate and screws)

**Supplementary Figure 3.** Funnel plot for publication bias with respect to comparison of pooled grip strength (as % of the unaffected side) among the two groups (i.e. pinning for metacarpal fractures compared to ORIF with plate and screws)

**Supplementary Figure 4.** Funnel plot for publication bias with respect to comparison of limb shortening (in mm) assessed by radiography among the two groups (i.e. pinning for metacarpal fractures compared to ORIF with plate and screws)
